# Citizens here and there against the crisis: immigrant organizations’ transnational solidarity during the COVID-19 pandemic

**DOI:** 10.3389/fsoc.2025.1519301

**Published:** 2025-03-21

**Authors:** Maurizio Artero

**Affiliations:** Department of Social and Political Sciences, Università degli Studi di Milano, Milan, Italy

**Keywords:** immigrant volunteering, COVID-19, immigrant organizations, transnationalism, solidarity, remittances, Italy

## Abstract

This article examines the solidarity initiatives undertaken by immigrant organizations in Italy during the COVID-19 pandemic, highlighting the significance of a transnational perspective in understanding these efforts. Drawing on two qualitative studies, particularly the analysis of 19 interviews with leaders of Italian immigrant organizations involved in solidarity work during the pandemic, the findings highlight that solidarity extends beyond co-ethnic support to encompass broader community engagement, especially in times of crisis. Indeed, the study reveals how immigrant organizations mobilized resources for both their home countries and the host society, demonstrating a commitment to collective wellbeing that transcends national boundaries. This article contributes to the discourse on transnational citizenship, revealing how immigrants enact substantive citizenship through participation in social initiatives that address urgent needs.

## 1 Introduction

In early 2020, COVID-19 quickly spread around the globe and caused a global pandemic, a situation that sparked a range of solidarity actions, protests, mutual aid efforts, self-mobilization, and self-organization, as well as exclusionary policies (Triandafyllidou, 2022). In particular, the pandemic prompted a surge in solidarity, both at the governmental level and among civil society. In the latter case, immigrants and their organizations provided tangible evidence of solidarity (IOM, 2020). In this paper, I look closely at the solidarity initiatives developed by immigrant organizations based in Italy during the pandemic.

The literature on immigrant organizations during the pandemic has predominantly adopted a homeland oriented or host-country-oriented perspective, neglecting to consider these ‘two sides’ together (e.g., Galam, 2020; Kynsilehto, 2020; Libal et al., 2021; Liu and Ran, 2020; Vilog and Piocos, 2021). Conversely, this paper advocates for the necessity of employing a transnational perspective on the actions of immigrant organizations during the pandemic. This approach entails examining the connections between various scales and contexts rather than merely viewing the country as a whole (Faist, 2018). Upon closer examination, it will indeed be noted that the activities performed by these organizations demonstrate a tendency to target both the receiving and sending countries. In particular, many immigrant groups, often organized by nationality, supported their homeland while also expressing solidarity within the host society, addressing both fellow immigrants and the native population (see also Artero and Ambrosini, 2024; Gatti, 2022; Wang and Li, 2024).

This argument stems from two exploratory studies I participated in between 2022 and 2024. The first was a research project examining the forms of aid and giving developed by foreign citizens in Italy during the COVID-19 pandemic; the second focused on the individual and collective remittances of immigrants in Italy. These research experiences allowed me to observe that, during the COVID-19 period, this transnational outlook functioned not only as an analytical concept but also as a moral stance. Other studies (e.g., Katsanidou et al., 2022) highlighted that the pandemic enhanced transnational solidarity in the EU, among states and citizens; here, I observe how the initiatives of the immigrant organizations under scrutiny represent an exemplary case of transnational solidarity, in the sense of cross-boundary social solidarity characterized by “feeling with” people struggling for the same issues. This concept of transnational solidarity is in accordance with Gould’s (2007), that is, a form of social empathy, with connections that bind individuals to distant others (individuals, social groups, associations). In this understanding, transnational solidarity can encompass social solidarity between individuals, communities, and organizations comprising members from disparate cultural backgrounds and national identities. Additionally, it can include identity-based relationships among dispersed members of a community.

As will be underscored, an important driver of many of these expressions of transnational solidarity is the sense of belonging to both their ‘sending’ and ‘receiving’ societies. I contend that this belonging can be interpreted as an expression of immigrants’ transnational citizenship that pushes them to civically engage in their homeland and the receiving context as well (see Smith, 2007).

## 2 Theoretical framework: transnationalism, transnational perspective and transnational citizenship

This paper uses a transnational perspective to look at the solidarity initiatives of immigrant organizations during the COVID-19 pandemic. Following Faist (2018), by transnational perspective, I refer not only to the traditional understanding of transnationalism (which refers to cross-border linkages) but to a wider reading “where ‘trans’ means the transgression of the national state and thus a perspective which does not take the national state as its point of departure but rather the nexus of various scales, ranging from the global to the local” (Faist, 2018, p. 22). Specifically, this perspective does not regard the nation-state as the primary analytical focus but scrutinizes the actual links across not only national borders but also within those borders (Faist, 2018, p. 22).

This perspective is all the more relevant given the current globalization processes. In particular, advancements in telecommunications and transport have enabled migrants to maintain identities and activities that simultaneously reference their places of origin and their host communities, effectively challenging the notion that they must be anchored in a single location (Ralph and Staeheli, 2011). In this connection, scholars have coined the term ‘transmigrant’ to describe individuals who move across international borders, settle, and establish connections in a new state, while maintaining ongoing social ties with their polity of origin (Schiller et al., 1992; Snel et al., 2006). Transmigrants’ identity and sense of belonging are tied to multiple places, and their lives systematically revolve around more than one nation-state (Goldring, 2002). In particular, transnational family relationships play a relevant role in these processes, as they favor interdependence between migrants and those left behind (Boccagni, 2015).

However, the feasibility of such a configuration of identity and belonging remains contested. Particularly, dominant political discourse often frames migrants’ efforts to maintain cultural and social ties with their country of origin as being at odds with their ability to ‘establish roots’ in the host country (Snel et al., 2006). In this sense, the emotional, and often also material, investment that migrants maintain with the motherland is perceived as a potential barrier to their full integration. For example, the sense of belonging and efforts to successfully integrate into the economic fabric of the receiving society—two important elements of integration (e.g., Ager and Strang, 2008)—may be threatened by transnational practices such as remittance sending (Joppke and Morawska, 2003; Levitt, 2003). However, empirical evidence does not support a clear-cut relationship between migrants’ transnational activities and their integration processes (Snel et al., 2006), and some researchers contend that these phenomena may be mutually reinforcing.

One reason is that transnational activities invariably require financial resources and are thus partly contingent on the economic capacity of the individuals involved (Al-Ali et al., 2001). For example, Mazzucato (2008) observes that migrants’ expenditures on their countries of origin—such as supporting families or investing in local projects—are often accompanied by efforts to establish their livelihoods in the host country. Additionally, Snel et al. (2006) observe that migrants’ identity generally revolves around identification with the inhabitants of one’s own original country, the identification with compatriots in the country of residence and, finally, the identification with the inhabitants in general of the residence country. These three identifications are not exclusive and can, and often do, co-exist. In this context, for many migrants, the identification is, arguably, ‘bifocal’, and migrants continuously negotiate identities, forging novel configurations of identification with the ‘new’ and the ‘original’ society.

These findings are consistent with the recent developments affecting the notion of citizenship. Unlike the traditional conceptualization of citizenship, predominantly associated with rights and responsibilities, historically embedded within national institutions, contemporary accounts of citizenship ‘expand the reach’ by focusing on citizenship’s ‘substantive dimensions.’ In particular, they emphasize not only citizenship’s formal dimensions linked to the legal status and rights but also dimensions like political activity and collective identity and sentiment, which bring the substantive character of citizenship to the fore (Bosniak, 2000).

Against this backdrop, it has been argued that ‘subjects’ become, in fact, citizens via political activities like protests and demonstrations (e.g., Isin and Nielsen, 2008). Simultaneously, as a collective identity and sentiment, contemporary conceptualizations of citizenship revolve around the affective ties of identification and solidarity (Lister, 2007). This approach brings forth the concept of ‘belonging’—personal, intimate, and emotional connections that encompass feelings of involvement and participation, and are characterized by their multiplicity, layers, and context-specific nature (Anthias, 2002). Therefore, while traditionalist views have often treated identity in an essentialist manner—as exclusive, singular, and cohesive—and tied to a single nation, the contemporary understanding tends to be more flexible and multifaceted (Antonsich, 2010; Colombo et al., 2009).

This conceptualization of citizenship helps us recognize how important citizenship dimensions are accessible to migrants both ‘within’ and ‘across’ nation-states. Indeed, within nation-states, migrants without the citizenship status of their country of residence can act to become citizens by engaging in political and civil activities (e.g., Bosniak, 2000; Shinozaki, 2015). For example, migrants can ‘act’ citizenship in their host society by engaging with local communities through civil society activities and routine practices (Staeheli et al., 2012; Theodore and Martin, 2007). In particular, even immigrants excluded from formal citizenship can engage in political actions (or even mere access to services), developing as a result a sense of belonging and emotional connections with their host society, while also claiming their ‘membership’, regardless of the formal status (Isin and Nielsen, 2008).

Additionally, we can appreciate how migrants tend to exercise citizenship prerogatives across nation-states, especially toward their home countries. Specifically, they practice substantive membership and citizenship in their communities of origin via practices like voting or civic initiatives. These initiatives also consist of social activities of a collective nature, including the initiatives of immigrant organizations (Goldring, 2002). Particularly, through these organizations, migrants can undertake activities that embody a broader vision of societal improvement, encompassing civil, social, and political progress, thereby enabling emigrants to function as ‘de facto citizens’ of their home communities (Goldring, 2002; Marini, 2013).

Participation in these groups can feed migrants’ identification with the original country, but this does not obliterate the possibility of participating and belonging to the host country. For example, in a study on Bolivian immigrants in Washington D.C., Strunk (2015) found that many immigrants in ‘diaspora’ organizations not only mobilize for their home country but also engage in political activism in Washington.

In this sense, and thanks to these groups, many migrants ‘possess’ a sort of transnational citizenship, acting as citizens and cultivating belonging in both original and host countries (Smith, 2007). In particular, many migrants forge novel identification configurations with the ‘new’ and the ‘original’ society (e.g., Appadurai, 1996). When investigating their sense of identity and belonging, they express a sense of belonging and allegiance to both ‘here’ and ‘there’ (Waters, 2009). For example, Snel et al. (2006) argue that the identity of many migrants revolves around both the identification with the inhabitants of one’s own original country, the identification with compatriots in the country of residence and, finally, the identification with the inhabitants in general of the country of residence. These three identifications are not mutually exclusive; they can, and often do, co-exist, representing the ‘two faces of transnational citizenship’ (Smith, 2007).

### 2.1 Defining transnational solidarity

In addition to citizenship, the notion of solidarity has undergone a process of transnationalization. From a sociological perspective, the traditional meaning of solidarity is hardly transnational. Indeed, it revolves around a relationship binding all the members of a single cohesive group or society;^1^ this conceptualization still stands, linking solidarity to the relations among individuals within a single group (usually thought of as a community), at most extended to individuals within a particular nation (see Schwiertz and Schwenken, 2020). To modernize this notion, social theorists have coined various notions of solidarity sensitive to relationships between individuals and others at a distance or to dialogical relations with others (Schwiertz and Schwenken, 2020). Against this backdrop, here I employ the notion of transnational solidarity by Gould (2007), where solidarity is a form of social empathy, or feeling-with, applying to intragroup relations across borders as well as to an individual’s relation to the members of a different group and the relations among groups. Other interpretations of transnational solidarity emphasize cooperation across borders or among people defined by shared characteristics (a kind of transnational but intragroup solidarity) (see Schwiertz and Schwenken, 2020). Gould’s take diverges from these conceptualizations; in it, transnational solidarity is not limited to specific others or identity-based groupings. Instead, it is open and inclusive toward individuals, communities, and organizations with different cultural backgrounds and national identities.

In her understanding, in particular, transnational solidarity emerges among “individuals who are concerned for each other and either do aid each other or recognize obligations to do so when necessary” (Gould, 2007, p. 153). Specifically, it is not only the solidarity that traverses space and countries, and unites people ‘at a distance’.

Rather, it is a ‘cross-boundaries’ social solidarity, in which the concerns of people in the same context but divided by social boundaries end up becoming your own (see also Wang and Li, 2024); an occurrence that finds an important articulation in ‘project-related solidarity,’ in which efforts are made to assist with a particular problem, like a disaster or a crisis.

This sentiment of transnational solidarity applies to individuals as well as collectives. As Gould (2007) asserts, solidarity can extend to relations among groups or associations. Accordingly, solidarity may exist among civil society associations, as well as between them and individuals and even institutions. In this connection, we can regard the initiatives of immigrant organizations during the pandemic as expressions of transnational solidarity. By immigrant organizations, I refer to civil society groupings whose members are foreigners; typically, they come from the same area (i.e., hometown associations) or the same nation-state (what I will call ‘homestate associations’ hereafter) and live in the same host country (Goldring, 2002).

During the pandemic, these organizations raised money among their members to finance collective remittances to their home countries. At the same time, they also expressed solidarity toward the host country. Their efforts exemplify transnational solidarity because immigrant organizations’ solidarity initiatives are based on the ‘transnational outlook’ of their leaders and members, a bifocal outlook involving both the home and host country. Simultaneously, they are also transnational inasmuch as they have cross-cultural backgrounds and national identities based on a sense of obligation and common concerns, adhering to Gould’s concept of transnational solidarity.

This analysis of the action of immigrant organizations during the pandemic (which juxtaposes a homeland oriented with a host country-oriented perspective) is partially consonant with Wang and Li′s work on Chinese immigrants in France during the pandemic (Wang and Li, 2024). Their contribution illustrates a solidarity movement initially oriented toward their home country that turned into a transnational solidarity movement; in particular, over time, the direction of solidarity shifted toward the host society and leaped ethnic borders and extended to the broader population. Consequently, the meaning of transnationalism gradually expanded from a transnational solidarity oriented toward the home country to a transnational solidarity focused on people differently positioned in terms of nationality and identity.

Unlike them, however, I will not refer to the notion of global citizenship when explaining the ‘forces’ behind these forms of solidarity, but instead to the notion of transnational citizenship. As will be seen, indeed, the expressions of solidarity of the participants have roots in their multiple belongings and their desire to exercise citizenship prerogatives in both the place where one lives and the place of origin.

## 3 Methodology

The materials informing this article originate from two qualitative studies. The first, in a chronological order, is a research project promoted by the ‘Italian Centro di Servizio per il Volontariato (CSV)’ on the forms of aid and giving developed by immigrant individuals^2^ in Italy during COVID-19. For this study, 65 in-depth interviews were conducted between January and July 2022 by a team of researchers (including the author of this paper) and CSV staff. The second study focused on the individual and collective remittances of immigrants in Italy. The research project involved administering a nationwide survey in Italy and conducting in-depth interviews. The second phase of the study was conducted between November 2023 and June 2024 and involved the participation of 60 individuals. In this case, I interviewed all the participants personally.

From these two studies, I selected 19 qualitative interviews: 12 from those conducted by the research team during the first investigation (identified as numbers 1, 2, 5, 6, 8, 9, 11, 15, 16, 17, 18, and 19 in Table 1) and 7 from the interviews of the second investigation (those with participants numbered 3, 4, 7, 10, 12, 13, and 14 in Table 1). In both investigations, participants were recruited using non-probabilistic sampling techniques, particularly snowball sampling. Consequently, this selection does not aim to achieve generalizability or representativeness, especially about the actions of the various national groups represented by the groups. However, the 19 interviews were chosen based on the prominence of the interviewee within the context of an immigrant organization, thus excluding those conducted with participants from ‘Italian’ associations or groups with a majority of Italian members, as well as those with members of immigrant organizations who do not hold leadership roles within them. Additionally, an effort was made to maximize the diversity of the participants’ national backgrounds. In this way, the aim is to shed light as comprehensively and in as much detail as possible on the activities of immigrant organizations based in Italy.

**Table 1:** List of interviewees.

Interview number	Name	Gender	Nationality	Role in organization
1	Freira	F	Argentina	President
2	Sorin	M	Romania	President
3	Diop	M	Senegal	Vice President
4	Hadi	M	Senegal	Administrator
5	Filip	M	North Macedonia	President
6	Zanita	F	El Salvador	Director
7	Costanza	F	Uruguay	Coordinator
8	Liliana	F	Cape Verde	President
9	Bayé	M	Burkina Faso	Vice President
10	Viola	F	Senegal	Administrator
11	Iffat	F	Tunisia	President
12	Marguerite	F	El Salvador	Founder/Ex-President
13	Habib	M	Senegal	President
14	Michaela	F	El Salvador	Representative
15	Evita	F	El Salvador	President
16	Mariel	F	Philippines	Representative
17	Maria Marta	F	Ecuador	President
18	Aamir	M	Morocco	Vice President
19	Asad	M	Tunisia	Coordinator

Following this selection procedure, this article is based on the qualitative analysis of 19 interviews with leaders of Italian immigrant organizations that, during the COVID-19 pandemic, engaged in solidarity work. By immigrant organization, I mean civil society groupings, more or less formalized, whose members are exclusively or in large majority immigrants (see Schrover and Vermeulen, 2005). These can be large and well-established, but they can also be small, informal, and unstable. The resulting sample includes a large set of organizations, comprising ‘diaspora’ associations (e.g., hometown and homeland organizations), which usually include only compatriots and immigrant international organizations, made up of immigrant people with different national origins. The characteristics of these organizations are detailed in Table 2.

**Table 2:** Organizations’ characteristics and their activities during the pandemic.

Interview number	Organization type	Location	Target community	Predominant activity in Italy	Predominant activity abroad
1	Mixed Women	Ancona	Immigrant Women	Psychological Support	
2	Homeland	Rome	Romanian	Food Distribution	In-Kind Donations
3	Homeland	Cosenza	Senegalese	Food Distribution	In-Kind Donations
4	Homeland	Venice	Senegalese	Fundraising to Italian Institutions	Financial Donations
5	Homeland	L’Aquila	North Macedonian	Food Distribution	Financial Donations
6	Homeland	Milan	Salvadoran	Financial Help	
7	Mixed Women	Milan	Mostly Latin American Women	Food Distribution	
8	Mixed Women	Naples	Miscellaneous/Mostly Cape Verde Women		Financial Donations
9	Homeland	Pescara	Burkinabè	Information Dissemination	In-Kind Donations
10	Homeland	Brescia	Senegalese	Information Dissemination	In-Kind Donations
11	Mixed Women	Palermo	Muslim Women	Food Distribution	
12	Homeland	Milan	Salvadoran	Financial Help	
13	Homeland	Milan	Senegalese	Food Distribution	
14	Homeland	Como	Salvadoran	Financial Help	Financial Donations
15	Mixed Women	Milan	Latin American Women	Food Distribution	
16	Homeland	Rome	Filipino	Information Dissemination	In-Kind Donations
17	Mixed Women	Rome	Latin American Women	Food Distribution	
18	Mixed Religious	Jesi	Muslim Immigrants	Food Distribution	
19	Mixed	Sondrio	Immigrants	Information Dissemination	Financial Donations

The selected interviews were all conducted in Italian and followed a semi-structured guideline with open-ended questions (Magnusson and Marecek, 2015). In the interviews related to the first study, topics included actions taken during the pandemic by the interviewees themselves and by the groups to which they belonged: activities, beneficiaries, targets, reasons to ‘take action,’ the outcome of this experience, and (where applicable) how their immigrant background affected their relationship with Italian beneficiaries, fellow volunteers, and organizations. In contrast, the interviews conducted for the second project focused on a range of topics, including the history and organizational structure of the guided group, the interconnection between migration and family remittances within the target community, collective remittances facilitated by the association, and perceptions of the efficacy of these.

Interviews were recorded and transcribed in Italian. In this process, participants were pseudonymized. Interview transcripts were subsequently translated into English only when directly quoted in this paper. The transcripts were coded and analyzed using thematic analysis (Braun and Clarke, 2006). Specifically, I employed an inductive research strategy aimed at identifying the most significant experiences and dimensions that emerged. After identifying regularly occurring experiences and dimensions, I introduced the procedure of analytical abduction to arrive at the findings. A qualitative analysis software, QDA Miner Lite, was employed to assist with the coding.

## 4 Transnational solidarity in Italy: solidarity across ethnic boundaries

It was 9 January 2020 when the World Health Organization announced that Chinese health authorities had identified a new strain of coronavirus, later renamed SARS-CoV-2. In Italy, the first indigenous case was reported on 21 February, followed by an escalation of cases, hospitalizations, and deaths. The government responded to these events with extraordinary measures, starting with a series of lockdowns, the longest and most important of which was established in March 2020 and continued for almost 2 months. Although these measures were likely necessary to contain the virus, they caused an economic downturn. These crises—both the health and economic ones—although suffered by the entire Italian population, particularly affected the immigrant population in Italy.

Firstly, despite the message that the virus does not discriminate, numerous studies have highlighted that migrants faced higher mortality and morbidity rates due to COVID-19 in various contexts, including Italy (Pagani et al., 2021). Additionally, the economic downturn caused by the pandemic disproportionately affected migrants, resulting in higher unemployment and poverty rates compared to native residents: over 1.5 million immigrants were in poverty in 2020, 29% of the total compared to 7.5% of Italian citizens (Quaranta et al., 2021).

Acknowledging these problems, several initiatives of immigrant associations emerged throughout the country, attempting to make up for institutional shortcomings (see Artero and Ambrosini, 2024; Gatti, 2022). In Table 2, I present a summary of the actions undertaken by immigrant organizations during the pandemic, outlining the predominant type of action. It emerges that 18 organizations (out of 19) provided help to the immigrant population in Italy. Forms of support include material (food, money) as well as ‘immaterial’ (mental help, information) help. This was primarily given to impoverished immigrants. Indeed, in a situation where the Italian state’s assistance to the most vulnerable people (including many immigrants) was deficient, they employed their (often scarce) economic and human resources to help the immigrants most in need. Hadi, head of a Senegalese association in Veneto, attests:

“*Among us, there were many people in difficulty: those who did not work, those who had lost their jobs. There were quite a few who were self-employed and who during COVID-19 were excluded from subsidies… We made bags of foodstuffs, which we delivered to people in need. And so did we in Vicenza, Verona, Padua, Venice… So, several of our groups responded, they found this form of help to be close to our compatriots in need.*” (Int.4)

As Hadi’s words suggest, many of these aids targeted the members of the organizations and subsequently their compatriots in general. This was particularly true for the homestate associations. Specifically, the predominant activities among these organizations were the distribution of foodstuffs and the collection and distribution of money. Many associations, in fact, decided to help their compatriots through donations aimed at meeting people’s basic needs, including bills and rent. In this context, Zanita says:

“*We helped our compatriots pay their rent and bills, because families, especially if they have children, have unavoidable needs.*” (Int.6)

However, it would be incorrect to characterize the efforts of immigrant organizations merely as forms of identity-based solidarity. Indeed, the interviewees stated that their aid often did not operate strictly within co-ethnic boundaries but depended on the social network of associations and their members, which often included immigrants of other nationalities. Immigrant associations, even when based on a singular nationality, often establish relationships with other immigrant organizations operating in the same locality. Simultaneously, many of these groups are more porous than expected, with organizations accepting members from other countries. For example, Salvadoran groups brought foodstuffs to Latin American families of different nationalities, and Senegalese groups also helped people from other parts of Africa.

In particular, crucial help extended by many organizations to the general immigrant population concerned information dissemination. During the pandemic, many government services, including immigration offices and courts, were unavailable. This situation affected the processing of visas, asylum claims, and other immigration-related applications, leaving migrants in a state of uncertainty about their legal status (e.g., Bonizzoni and Artero, 2023). Specifically, interviewees recounted problems with the renewal of residence permits or how to participate in the regularization program that the Italian government introduced in June 2020, shortly after the COVID-19 outbreak (see Bonizzoni et al., 2021). Some of the organizations thus acted to provide bureaucratic information or put people in contact with institutions (e.g., Bonizzoni and Artero, 2023). Others engaged in providing information on access to health services, including vaccination campaigns (see Artero and Ambrosini, 2024). Due to healthcare access issues, immigrants were more exposed to SARS-CoV and had trouble getting COVID-19 swab testing (Profili et al., 2022), especially when undocumented. Accessing healthcare services was a significant challenge; obtaining reliable information about the pandemic and available services, including testing and vaccination programs, was also a problem (Pagani et al., 2021). Additionally, forms of help for specific ‘categories’ of immigrants, representing diverse nationalities, have emerged. A notable case relates to solidarity among women. Among the associations of the participants, some were founded by women of various nationalities with the intention of helping foreign women in general, regardless of their origin. Sadly, during the pandemic, the social distancing measures, while necessary, heightened feelings of isolation among immigrants who were cut off from their cultural and support networks—a condition particularly taxing for immigrant women (Dotsey et al., 2023; Quaglia and Tognetti, 2020). In response, some of these organizations offered psychological support and ‘virtual’ proximity to women who suffered from the social effects of the pandemic or raised awareness of the problems of domestic violence, a phenomenon that increased during the pandemic (Sabri et al., 2020).

Consequently, it is difficult to separate the assistance that developed solely within identity-based solidarity from solidarity open to individuals, communities, and organizations with different cultural backgrounds and national identities. In this context, the participants also provided examples of solidarity offered to the general society. Numerous examples of aid to the broader Italian society exist, starting with those aimed at its institutions. From this perspective, a noteworthy contribution from organizations concerned the collection of funds and materials to be allocated mainly to public institutions, such as hospitals or local administrations (see also Artero and Ambrosini, 2024).

“*In the last two years, we have done a lot. One example: we collected money and gave it to the region. And we made another payment to the civil protection. In addition, we assisted many Italians. This shows that our efforts extend beyond our 'community' and are directed towards helping others.*” (Int.4)

Furthermore, instances were observed of associations assisting Italian citizens experiencing economic difficulties as part of the social network of the associations and their members (in a manner analogous to that noted with non-compatriot immigrants in Italy). Among these forms of solidarity, however, perhaps the most emblematic is the participation of some associations in activities promoted by municipalities or local authorities. A case in point is Diop’s association. Diop is the vice president of an association established in 2016 as a ‘homestate’ association of Senegalese migrants in the area of Cosenza, Calabria. This association participated in the home deliveries promoted by the municipality of Rende, a city close to Cosenza. Diop’s association was not the only immigrant organization to join this initiative and distribute food to many (usually Italian) people in need in Rende.^3^ These examples illustrate how ‘immigrant’ associations often have the ambition to contribute to the general wellbeing, with initiatives that coexist with those dedicated to their own ‘ethnic community’ in Italy.

## 5 Collective remittances and solidarity at a distance

Though Italy was among the first countries severely affected by COVID-19, the virus’s spread became a true global experience. On 11 March 2020, the World Health Organization (WHO) officially declared that the COVID-19 outbreak had evolved into a pandemic, indicating that the disease had spread worldwide.

In particular, the COVID-19 pandemic had significant repercussions on the ‘sending countries’ of immigrants in Italy: job losses, economic slowdowns, and healthcare system struggles affected many immigrants’ home nations (Bong et al., 2020; Josephson et al., 2021). Consequently, immigrants endured the effects of the COVID-19 pandemic in two contexts: directly, as Italian residents, and vicariously, through the problems affecting their homelands and their loved ones there. They specifically faced difficulties and anxieties tied to their homelands without the possibility (at least initially) of returning home for critical reasons, such as the illness or death of a family member, due to restrictions on international human mobility, putting a severe psychological burden on them (Quaglia and Tognetti, 2020). Moreover, many immigrants, normally responsible for supporting their families back home through remittances, during the COVID-19 economic crisis were unemployed and unable to send money to their homelands. This meant not only a personal financial plight but also a crisis for the families in their home countries, who were already suffering from the economic downturn (Remittance Community Task Force, 2020).

Against this backdrop, the interviews revealed that 10 organizations managed to provide some form of help to their members’ sending countries despite numerous difficulties (Table 2). As shown in Table 2, these groups sent financial and in-kind donations. Specifically, these supports represent transnational solidarity actions under the guise of collective remittances.

Collective remittances are money or goods raised by an immigrant group and sent to the homeland to benefit a group or a community beyond family ties (Goldring, 2002). Although scholarship on collective remittances has mainly investigated remittances coming from formal and organized associations in response to governments’ demands, it has been shown that informal collectives whose agendas are unconnected with homeland governments are the most important source of collective remittances (Galstyan and Ambrosini, 2022).

The economic consequences of COVID-19 on their homeland compelled immigrant organizations to organize fundraising among their members. In this regard, it is paradigmatic of what Liliana did with her association of Cape Verdean women in Naples, raising money to help needy people in Cape Verde:

“*Especially with the COVID-19, my island, Cape Verde, being a tourist island, suffered a lot, especially economically, and so many families found themselves in difficulty. We collected money because there is a lot of help from the immigrant network. So, we did this fundraising for the neediest families in Boa Vista.*” (Int.8)

As in Liliana’s case, in the overwhelming majority of instances, the preferred form of homeland-oriented solidarity is through monetary collective remittances via fundraising for people in need. However, it was not just a matter of sending money but also basic necessities, as in the case of Sorin and Diop, leaders, respectively, of a Romanian association in Rome and a Senegalese organization in Cosenza, who sent foodstuffs to people they knew were in serious financial difficulty.

As in the aforementioned cases, the sending of materials or money was not something extemporaneous but was part of the collective remittances that these immigrant organizations had been sending even before the COVID-19 pandemic. In particular, for homestate associations, the collection, management, and distribution of collective remittances are crucial aspects of their actions (see also Galstyan and Ambrosini, 2022). From this perspective, these groups exemplify how migrants’ solidarity activities during health crises are often in continuity with already existing initiatives (see Rubyan-Ling, 2019). However, there are also instances of ‘mixed’ or ethnic associations that organized international collective aid for the first time during the pandemic. In this context, sending goods during the pandemic was challenging. Often, participants expressed their desire to help their home countries but were unable to do so because of two main reasons: the financial fragility of their organizations and the restrictions and difficulties concerning the sending of in-kind remittances. Regarding the first issue, many associations formed by individuals with an immigrant background have been forced to suspend many of their traditional activities, first due to confinement and then due to restrictions on gatherings in Italy during the pandemic emergency. These organizations have particularly suffered from the worsening economic conditions of many of their members, who have consequently allocated fewer resources to them; they have also stopped activities such as face-to-face fundraising and have been affected by other sources of income to support associations. All of this has exacerbated the situation of the immigrant organizations that often decided to privilege the needs of their members in Italy.

In addition, there were restrictions and difficulties concerning international mobility that made it more difficult to send materials. A case in point is the one presented by Bayé, who helped organize the sending of materials by private plane to Burkina Faso despite the suspension of flights and transport to the country.

“*I collaborated with the Neapolitan section of my association when they decided to organize a plane to take goods to Burkina Faso, because no more planes were going there… The president thus had to hire a plane to bring medicines and other goods.*” (Int.9)

However, as seen, the dawning realization of the seriousness of the problems led organizations and community groups to make significant efforts to aid communities at home. In these cases, I contend that these examples of transnational solidarity share important features with what have been called initiatives of “diasporic humanitarian mobilization” (Rubyan-Ling, 2019). With this term, Rubyan-Ling defines the aid provided by diaspora associations (e.g., hometown and homeland organizations) toward their homeland. Their work differs from the traditional humanitarian work of big international organizations during acute crises.

In contrast to their professionalized, ‘altruistic’, neutral, and expert-led help, diasporic organizations act more out of a concrete sense of ‘embeddedness’ to a particular ‘homeland’: these organizations are usually ‘rooted’ in their homeland, and an ethic of familial and known-community obligation nourishes their efforts (Rubyan-Ling, 2019). Additionally, collective remittances (especially when emerging from ‘below’) develop within social bonds. As argued by Galstyan and Ambrosini (2022), trust, connectedness, and reputation facilitate immigrant organizations to identify beneficiaries and organize the delivery of material and immaterial goods. To illustrate these points, I put forward the case of Viola.

Viola is a woman in her mid-20s who lives in Bergamo. She arrived in Italy following her father when she was about 6 months old and has worked and lived in Italy most of her life. In 2020, during the critical moments of COVID-19, she and other young people of Senegalese origin decided to set up an association, with the initial objective of helping children studying in Koranic schools in Senegal who were in precarious conditions during COVID-19.

The birth of the association is an example of improvisation but also transnational solidarity. The group was formed from a live broadcast on Instagram that another Italian-Senegalese young man, the future president of the group, was transmitting; in this live broadcast, the future president, stuck in Senegal because of the restrictions on international mobility, explained the COVID-related difficulties experienced by Senegalese kids. The participants decided that they could take concrete action by agreeing to send at least 5 euros each to the president so that he could buy medical supplies, especially for students in Koranic schools in Senegal. Subsequently, the president proposed setting up an association, which then became formalized and distributed more than 500 COVID-19 prevention kits to students. The impetus, therefore, came from learning about the difficulties in the country of origin. As Viola states,

“*At the beginning, we were so frustrated to see all this… our main goal, what would make us really proud, was to improve these situations with our contribution, as much as small it might be.*” (Int.10)

The activities of this association during COVID-19 were initially managed by the president, who was present in Senegal, and later (upon his return to Italy) by volunteers living in Senegal who distributed aid. After COVID-19, however, the association continues to operate in both Senegal and Italy, expanding its services thanks to its sponsor members, all young Senegalese individuals residing in Italy. Nonetheless, the association has roots in Senegal because it operates through volunteers who are there and also because its members feel emotionally connected to this country:

“*The main driving force for me, and I believe for others too, is the love for the land of our parents and the people there.*” (Int.10)

Moreover, the desire and expectation of transnational mobility also appear to offer an impetus for this solidarity effort. Viola argues that:

“*This is a common desire of many members, who hope one day to be able to go to Senegal to live there… Personally, and for other group members I know, the goal is to return to our country within the next five to ten years and start a business.*” (Int.10)

## 6 Help across national and ethnic borders: forms of emergency-driven transnational citizenship

These experiences were fueled by the realization that the emergency was affecting people across national and ethnic borders. This realization seems to have ‘transcended’ the dimensions of belonging and citizenship among the members of the organizations and enhanced their willingness to help those in need in the two ‘directions’ described. In the case of immigrants’ efforts toward the ‘homeland’, there was specifically a sense of frustration and discomfort at seeing the difficult situation of people in the ‘homeland’. In Viola’s previous case, for example, she explained that the driving force behind her and the other members’ commitment was the discovery of children’s difficulties in Senegal, their “place of origin,” to which they have a deep emotional attachment. Hers was not the only case; Asad, for example, before he was able to send help through his association, decided to return to Tunisia and assist the people in his homeland. His words testify not only to his desire to help his original country during a COVID-related crisis but also to the difficulties these organizations faced in aiding people ‘abroad’:

“*In 2021, there was a strong spike [of COVID-19] in Tunisia. I tried to make some donations with my association, but because of too much bureaucracy and paperwork, the time was running out. So, I took a flight and went there as a volunteer.*” (Int.19)

Similarly, what drives the actions directed toward Italy—including both compatriots and immigrants in general as well as the ‘mainstream society’—is the desire to address the problems the Italian state seemed unable to manage. Consequently, these organizations especially provided help to defend the most vulnerable individuals from economic problems, often without discriminating between their members, compatriots, or Italian citizens, as previously observed.

“*I felt this need because I saw that people had needs, and I tried to help them.*” (Int.8)

“*I was welcomed in Italy, and it would be great if everyone could experience that same warmth and hospitality, like I did in Palermo. Here, we help each other out, no matter where you come from. I've noticed that many Italian people help immigrants, and also that immigrants help Italians, as we did. This was especially important during the pandemic, when many people needed help, food, and support. It's in these situations that you realize that everyone is vulnerable at some point and that everyone has the potential to be strong when it comes to helping others.*” (Int.11)

As this last interview with Iffat revealed, some interviewees feel grateful for the opportunities they have found in Italy and wanted to ‘return’ the favor during the emergency induced by the pandemic. In this sense, as argued by Gould (2007, p. 153), transnational solidarity emerges among individuals who care for each other and recognize obligations to assist one another when necessary. At the same time, for many, there is also a sense of identification and responsibility toward the place where one lives. If integration is also about feeling part of the new ‘host society’ (Ager and Strang, 2008), the examples of solidarity directed toward Italy demonstrate a certain degree of integration among the members of these groups (see also Ambrosini and Artero, 2022). This element, then, coexists with the desire to assert their full belonging to Italy (see also Artero and Ambrosini, 2024). In this regard, Iffat also states:

“*When I heard about immigrants who ‘do nothing for the society they come from,’ ‘they come and take but don't give anything,’ then I wanted to prove that this is my land too and I have to contribute to it…*” (Int.11)

As evidenced in this last excerpt, issues of citizenship and belonging frequently emerge during the interviews. Often, participants maintain that they are ‘de facto’ Italian citizens and possess a sense of belonging to Italian society. This sense of belonging is not exclusive: many indicated that they consider themselves ‘substantive’ Italian citizens, even when they are not by law, as well as citizens of their ‘original country’. Their understanding aligns with the definition of transnational citizenship, whereby transnational citizens have a conception of belonging that transcends a singular context and affiliation (Smith, 2007). Asad eloquently states:

“*In Italy, I completed my training, found work, and was able to start a family. Now there is me, my wife, my kids, my in-laws, and a great group of friends and acquaintances. […] I've got two pairs of glasses now, not just one. I'm Tunisian by origin and Italian by adoption, so I see the world through double lenses.*” (Int.19)

The interview excerpts we have seen should not be misinterpreted. Even though it is the leaders of the organizations who speak, the motives behind the solidarity actions appear to reflect the will of their members. In this regard, the interviewees indicate that decisions regarding the provision of assistance were frequently made in consultation with members. In some instances, this involved deliberations on the rationale for organizations to offer support. Thus, like Viola, who spoke of a shared sense of belonging to Senegal as a motive for engagement, other interviewees highlighted dimensions of citizenship and belonging as elements of a collective drive for solidarity. In this sense, the ‘bifocality’ of the members of the organizations seems to have played an important role.

This bifocality involves both the country of origin and the country of destination and appears to contradict the concerns of those who view a commitment to the country of origin as being at odds with the country of residence, and vice versa. In this connection, it is noteworthy to consider that as many as nine organizations were engaged in solidarity initiatives in Italy and their country of origin simultaneously. In instances where this was not the case, the primary reason was the difficulty in helping the country of origin, as previously discussed. Otherwise, it appears that the desire to engage with and contribute to the societies with which one identifies plays a pivotal role. In the case of Hadi and Habib, for example, the desire to provide assistance to both the country of origin and the host country originates from a common root of membership:

“*If one feels a country is his own, it is normal to do something to help.*” (Int.4)

“*[In helping Senegal or Italy] The motive is the same, in the sense of being able to do something to improve these societies.*” (Int.13)

Ultimately, these interviews show examples of transnational solidarity that traverse space and countries as well as social boundaries within countries. In these cases, the feeling of membership in both Italian and original societies fuels the desire and feeds the obligations to help ‘your societies’ during a critical moment.

## 7 Conclusion

This study of the solidarity initiatives developed by immigrant organizations based in Italy during the pandemic illustrates the importance of examining the COVID-19 pandemic from a transnational perspective. The initiatives of the immigrant organizations here under scrutiny, indeed, represent an exemplary case of transnational solidarity, in the sense of cross-boundaries social solidarity characterized by “feeling with” individuals struggling with the same issues. In particular, I employed Carol Gould’s conceptualization of transnational solidarity. According to her, the transnational one is a form of solidarity that transcends space and countries and unites people ‘at a distance’, as well as a form of ‘cross-boundaries’ social solidarity, in which also the concerns of people in the same context, but divided by social boundaries, end up becoming your own. In this contribution, I note that during the pandemic, immigrant organizations in Italy raised funds among their members to finance collective remittances to their home countries. At the same time, they also expressed solidarity toward the host country.

In particular, firstly, I observed several initiatives of immigrant associations that attempted to make up for institutional shortcomings by providing help to the immigrant population in Italy, as well as examples of solidarity provided to the general local society, starting with those aimed at its institutions. I argue that immigrant organizations demonstrated various forms of assistance that not only developed within co-ethnic lines but, in some instances, showed solidarity extending toward the local community and crossing ethnic boundaries to benefit the wider population. Consequently, they show how solidarity can be open to individuals, communities, and organizations with different cultural backgrounds and national identities. Secondly, it highlighted how the COVID-19 pandemic represented a true global experience. In particular, the COVID-19 pandemic had significant repercussions on ‘sending countries’ of immigrants. Against this backdrop, participants disclosed that their organizations managed to provide some form of help to the ‘sending countries’. These groups, specifically, organized transnational solidarity actions under the guise of collective remittances; these were the sending of resources (money or goods) emerging out of a concrete sense of ‘embeddedness’ to a particular ‘homeland’. Finally, to account for these dynamics, I especially refer to the notion of transnational citizenship. In particular, immigrant organizations’ solidarity initiatives are based on the ‘bifocality’ of their leaders and members, a bifocality involving both the home and host country. Specifically, many immigrant groups, often organized based on nationality, supported their homeland but also expressed solidarity within the local society, targeting both fellow immigrants and the native population too.

In particular, for many, these forms of transnational solidarity emerge from a sense of identification and responsibility toward two ‘societies’ at the same time. It is frequently the case that the solidarity initiatives undertaken by immigrant organizations are based on the ‘bifocality’ of their leaders and members, comprising both the home country and the country of destination. Ultimately, their efforts are examples of transnational solidarity because immigrant organizations’ solidarity initiatives are based on the ‘transnational outlook’ of their leaders and members—a bifocal perspective that incorporates perspectives involving both home and host countries. Simultaneously, they are also transnational since they traverse cultural backgrounds and national identities, founded on a sense of obligation and common concerns. In particular, I point out that to feed this solidarity, there was the realization of an emergency affecting people across national and ‘ethnic’ borders. In this sense, I noted how nine organizations were simultaneously engaged in solidarity initiatives in Italy and the country of origin. For the leaders of these organizations, specifically, the desire to assist the country of origin and the host country can be seen to originate from a common root of membership as well as obligations to help ‘your societies’ during a critical moment.

Against this backdrop, the interviews also highlight another aspect related to the issue of integration. As I noted at the end of the previous section, members appear to mobilize their organizations due to a sense of belonging to both their original context and the one in which they currently reside. This contrasts with the fear expressed in dominant political discourses that integration and transnational behavior are mutually exclusive (Snel et al., 2006). If integration encompasses a sense of belonging to a collective identity, this ‘double belonging’ may indicate a certain degree of ‘integration’ alongside an aspiration to engage in both contexts. In this regard, the pandemic offered an opportunity to demonstrate this by acting as de facto citizens in both their original and resident countries.

To summarize, in this contribution, I observed that whereas the bulk of the literature on immigrant organizations during the pandemic predominantly employed either a homeland-oriented or a host country-oriented perspective, without considering these ‘two sides’ together, here I adopt a transnational perspective that integrates these two aspects. In this way, I consider the ‘two faces of transnational citizenship’ of immigrants in Italy (Smith, 2007). As this contribution illustrates, indeed, immigrants ‘act’ citizenship in their host society as well as citizenship prerogatives toward their countries of origin through these organizations. Specifically, they practice forms of ‘substantive citizenship’ based on participation in collective life and multiple belongings that transcend simplistic ethnic or national labels and potentially materialize in different contexts, regardless of one’s legal-formal status (Isin and Nielsen, 2008). In particular, the transnational citizenship emerging from these initiatives originates from a sense of belonging to multiple contexts, which unravels across borders (Faist, 2018), prompting migrants to act and claim citizenship in multiple locations. In conclusion, it should be noted that the analysis presented here is subject to some limitations. It is based on data collected in two distinct exploratory research projects. To better articulate and corroborate the observations, it would be beneficial to conduct an explanatory study that delves into the reasons why transnational solidarity seems to emerge in relation to the pandemic. Furthermore, additional studies on the initiatives of immigrant organizations based in other national contexts would help to understand the generalizability of what has been observed here.

## Data Availability

The datasets presented in this article are not readily available because of the right to protection of personal data. Requests to access the datasets should be directed to the author.

## Footnotes

^1^In the usage of Durkheim (2016), solidarity pertains, initially, to the relation among members of traditional communities where each member is similarly characterized in terms of identities and perspectives, and, later, expands to people linked in interdependent relations with others through an extended division of labor.

^2^With this term, I include first and second-generation migrants.

^3^ https://www.comune.rende.cs.it/news/avvisi/2020/03/23/elenco-associazioni-volontari-per-consegna-gratuita-adomicilio-generi-alimentari-951/
